# Anti-inflammatory Effects of Abdominal Vagus Nerve Stimulation on Experimental Intestinal Inflammation

**DOI:** 10.3389/fnins.2019.00418

**Published:** 2019-05-08

**Authors:** Sophie C. Payne, John B. Furness, Owen Burns, Alicia Sedo, Tomoko Hyakumura, Robert K. Shepherd, James B. Fallon

**Affiliations:** ^1^Bionics Institute, Fitzroy, VIC, Australia; ^2^Medical Bionics Department, University of Melbourne, Parkville, VIC, Australia; ^3^Florey Institute of Neuroscience and Mental Health, Parkville, VIC, Australia; ^4^Department of Anatomy and Neuroscience, University of Melbourne, Parkville, VIC, Australia; ^5^Department of Otolaryngology, University of Melbourne, Parkville, VIC, Australia

**Keywords:** vagus nerve stimulation, peripheral nerve stimulation, inflammatory bowel disease, medical device, bioelectric neuromodulation

## Abstract

Electrical stimulation of the cervical vagus nerve is an emerging treatment for inflammatory bowel disease (IBD). However, side effects from cervical vagal nerve stimulation (VNS) are often reported by patients. Here we hypothesized that stimulating the vagus nerve closer to the end organ will have fewer off-target effects and will effectively reduce intestinal inflammation. Specifically, we aimed to: (i) compare off-target effects during abdominal and cervical VNS; (ii) verify that VNS levels were suprathreshold; and (iii) determine whether abdominal VNS reduces chemically-induced intestinal inflammation in rats. An electrode array was developed in-house to stimulate and record vagal neural responses. In a non-recovery experiment, stimulation-induced off-target effects were measured by implanting the cervical and abdominal vagus nerves of anaesthetized rats (*n* = 5) and recording changes to heart rate, respiration and blood pressure during stimulation (10 Hz; symmetric biphasic current pulse; 320 nC per phase). In a chronic experiment, the efficacy of VNS treatment was assessed by implanting an electrode array onto the abdominal vagus nerve and recording *in vivo* electrically-evoked neural responses during the implantation period. After 14 days, the intestine was inflamed with TNBS (2.5% 2,4,6-trinitrobenzene sulphonic acid) and rats received therapeutic VNS (*n* = 7; 10 Hz; 320 nC per phase; 3 h/day) or no stimulation (*n* = 8) for 4.5 days. Stool quality, plasma C-reactive protein and histology of the inflamed intestine were assessed. Data show that abdominal VNS had no effect (two-way RM-ANOVA: *P* ≥ 0.05) on cardiac, respiratory and blood pressure parameters. However, during cervical VNS heart rate decreased by 31 ± 9 beats/minute (*P* ≥ 0.05), respiration was inhibited and blood pressure decreased. Data addressing efficacy of VNS treatment show that electrically-evoked neural response thresholds remained stable (one-way RM ANOVA: *P* ≥ 0.05) and therapeutic stimulation remained above threshold. Chronically stimulated rats, compared to unstimulated rats, had improved stool quality (two-way RM ANOVA: *P* < 0.0001), no blood in feces (*P* < 0.0001), reduced plasma C-reactive protein (two-way RM ANOVA: *P* < 0.05) and a reduction in resident inflammatory cell populations within the intestine (Kruskal–Wallis: *P* < 0.05). In conclusion, abdominal VNS did not evoke off-target effects, is an effective treatment of TNBS-induced inflammation, and may be an effective treatment of IBD in humans.

## Introduction

Inflammatory bowel diseases (IBDs), encompassing Crohn’s disease and ulcerative colitis, are progressive debilitating immune-mediated disorders of the gastrointestinal tract (Ananthakrishnan, 2015b). The impact of the disease on patient quality of life is substantial due to its onset in young adulthood, fluctuating periods in which the disease is active (relapse and remission) and the lack of a cure (Abraham and Cho, 2009). The incidence of IBD is on the increase worldwide, with the prevalence of the disease highest in North America with an estimated 1.5 million people affected (Ananthakrishnan, 2015b).

The etiology of IBD is unknown, however, interactions between an individual’s genetic makeup and external environment (i.e., diet, stress) play key roles in the emergence of IBD (Ananthakrishnan, 2015a). IBD is characterized by the over production of the key upstream pro-inflammatory mediator tumor-necrosis-factor-alpha (TNF-α) from macrophages, monocytes and differentiated T cells within the gastrointestinal tissue (Sanchez-Munoz et al., 2008). The production of TNF-α leads to the infiltration of inflammatory cells, which themselves further release pro-inflammatory cytokines, such as interleukin-1β (IL-1 β), IL-6, and interferon-gamma (IFN-γ) (Sanchez-Munoz et al., 2008; Neurath, 2014). Current gold standard immunosuppressant pharmacological therapies and anti-TNF-α biologicals, suppress the immune reaction and reduce the cascade of cytokine release by targeting TNF-α production (Rutgeerts et al., 2004; Nielsen and Munck, 2007). Although clinical management of such combined therapies has led to advancements in the control and prediction of the disease (Caprilli et al., 2006; De Cruz et al., 2015), adverse events in response to medication can be experienced in up to 20% of IBD patients when using these therapies (Chaparro et al., 2013; Gecse et al., 2016). Furthermore, despite the development of new anti-TNF-α therapies and clinical management strategies, surgical resection of the inflamed area of the gastrointestinal tract is necessary in 80% of ileocecal Crohn’s disease patients (Caprilli et al., 2006). Therefore, an alternative therapy that keeps patients in remission is needed to more effectively treat IBD over the long-term.

A growing body of evidence has demonstrated that unilateral electrical stimulation of the left cervical vagus nerve is a feasible treatment in a rodent model of colitis (Meregnani et al., 2011; Sun et al., 2013). Following chemically-induced colitis in rats, cervical vagus nerve stimulation (VNS) improved the disease activity index (DAI: weight and stool quality), decreased histological damage and reduced inflammatory molecular markers expressed in colonic tissue (Meregnani et al., 2011). However, the effects of VNS therapy on histology and molecular markers were only seen in areas adjacent to, but not within, the inflammatory lesion. In a subsequent colitis study, inflammatory markers were only modestly reduced by cervical VNS, and inflammatory disease parameters (DAI, histology, inflammatory cytokine production) in VNS treated tissue did not return to control levels (Sun et al., 2013).

A first, small clinical trial demonstrated efficacy cervical VNS in ileocecal Crohn’s disease patients (Bonaz et al., 2016) The majority of patients (5 of 7) responded to treatment and showed a reduction in clinical symptoms (Crohn’s DAI), improvements in molecular markers (C-reactive protein and fecal calprotectin) and endoscopy DAI score. However, two patients experienced worsening of clinical symptoms and were removed from the study (Bonaz et al., 2016). Additionally, patients reported voice alterations (dysphonia) during stimulation (Bonaz et al., 2016). Other side effects, such as coughing, pain and labored/shortness of breath (dyspnea) are frequently reported following cervical VNS in patients treated with VNS for drug resistant epilepsy (Ben-Menachem et al., 2015). Studies in epileptic children fitted with a cervical VNS report more serious complications during sleep. Stimulation-induced effects on respiration and a reduction in overall oxygen saturation was seen in the majority of patients (87.5%, 8 patients; 100%, 10 patients, respectively) (Nagarajan et al., 2003; Zaaimi et al., 2005), while stimulus-induced changes to heart rate variability are reported in 50% of patients (10 patients) (Pruvost et al., 2006; Zaaimi et al., 2007). Another study reports stimulus-induced obstructive sleep apnea (15%; 26 patients) (Khurana et al., 2007). Such off-target affects are due to the activation of low threshold cervical vagal fibers to the larynx, pharynx, heart, and lungs (discussed in detail in the discussion), while the abdominal vagus nerve is at a site below these branches and its stimulation is predicted to results in fewer off-target effects.

**FIGURE 1:**
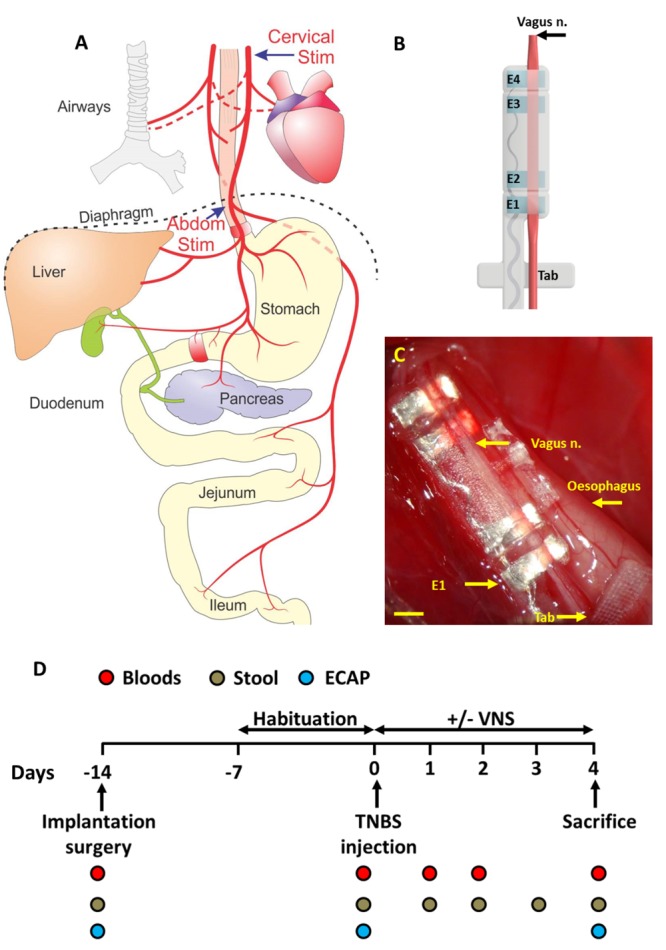
Abdominal vagus nerve anatomy, electrode design and experimental schedule. **(A)** Schematic anatomical diagram shows the cervical and abdominal branches of the vagus nerve. Off-target effects in response to cervical stimulation (Cervical Stim indicated by arrow) and abdominal stimulation (Abdom Stim indicated by arrrow) were evaluated. For the VNS efficacy experiment, the electrode array was implanted onto the anterior abdominal vagus nerve, below the diaphragm and above the hepatic and celiac vagal branches. **(B)** The cuff electrode array had two platinum electrode pairs (E1–E2; E3–E4) that stimulated and recorded evoked neural responses. The array was anchored by suturing the Dacron tab to the esophagus. **(C)** Image of the abdominal vagus nerve electrode array *in vivo*. **(D)** Experimental schedule for the VNS efficacy experiment. Scale bar in **(C)** 1 mm.

Although evidence for VNS therapy to treat IBD is promising, the current approach of stimulating the cervical vagus nerve has a number of clinical limitations, including potentially an undesirable side-effect profile. To overcome the limitations of cervical VNS, we hypothesized that stimulating the sub-diaphragmatic abdominal vagus nerve (Figure 1A), which is below vagal branches to the lungs and heart and closer to the end organ, will improve the therapeutic effect of VNS and have fewer off-target effects. To address this hypothesis, in this study we first developed an electrode array (in house) that was able to stimulate and record neural responses from the vagus nerve of the rats. Using this electrode array in a non-recovery experiment, referred to as the “*VNS off-target experiment*,” off-target effects to cardiac and respiratory rate were assessed during abdominal and cervical VNS. In a recovery experiment, referred to as the “*VNS efficacy experiment,”* the efficacy of abdominal VNS was assessed using a rodent model of chemically-induced intestinal inflammation (Figure 1A). Behavioral, molecular and histological markers of inflammation were evaluated to determine the efficacy of abdominal VNS, and the histology of the implanted vagus nerve examined to confirm the safety of electrode array and stimulation delivered.

## Materials and Methods

This paper describes: (i) an acute (non-recovery) experiment that evaluated off-target effects during cervical and abdominal VNS. This is referred to as the “*VNS off-target experiment*”; and (ii) a chronic VNS efficacy experiment that evaluated the efficacy of abdominal VNS following chemically-induced intestinal inflammation, which is referred to as the “*VNS efficacy experiment*” The methods section describes general procedures common to all experiments, followed by techniques specific to the VNS off-target and efficacy experiments.

### General Methods

#### Animals and Anaesthesia

Sprague-Dawley rats (10–12 weeks old, Animal Resource Centre, Western Australia) were used, and all animal procedures were approved by the Animal Research and Ethics Committee of the Bionics Institute and complied with the Australian Code for the Care and Use of Animals for Scientific Purposes (National Health and Medical Research Council of Australia). Approval was also obtained from the United States Army Medical Research and Material Command Animal Care and Use Review Office, protocol SSC-7486.02. Animals were kept on a 12 h light (7 a.m.–7 p.m.)/dark cycle (7 p.m.–7 a.m.) and allowed *ad libitum* access to fresh food, standard chow and water. For all surgical interventions, rats were anaesthetized (2–3% isoflurane using an oxygen flow rate of 1–1.5 L/min) and breathing rate remained between 45 and 60 breaths per minute (Payne et al., 2018c). All procedures were performed under aseptic conditions and following recovery surgery, rats were monitored carefully, given an analgesic (Carprofen 5 mg/kg sub-cutaneous) and housed separately. At the conclusion of the experiment, rats were anaesthetized (2% isoflurane using an oxygen flow rate of 1–1.5 L/min), then euthanized (300 mg/kg Lethabarb, intracardial injection).

#### Design of the Vagus Nerve Electrode Array

The vagus nerve electrode array consisted of four platinum (99.95%) electrodes embedded in a medical grade silicone elastomer cuff. Each platinum electrode had an exposed surface area of 0.3 mm^2^. The distance between adjacent electrodes (E1–E2, or E3–E4, center to center) was 1.2 mm, while the distance between electrode pairs (E1–E2 to E3–E4, center to center) was 4.7 mm (Figure 1B). A channel (0.55 mm wide × 0.2 mm deep) traversed the length of the array to position the vagus nerve in close contact with the electrodes without damaging the nerve. A silicone ‘lid’ completes the cuff, preventing the nerve from migrating from the channel. A Dacron embedded silicone tab adjacent to the electrode array was used to suture the array to the esophagus, when chronically implanted onto the abdominal vagus nerve, in order to provide mechanical stability (Figure 1B). Individually insulated 50 μm diameter platinum/iridium (90/10) wires were welded to each electrode and formed a helical cable which traversed to a percutaneous connector mounted on the lumbar region of the rat.

#### Abdominal Vagus Nerve Electrode Array Implantation Surgery

Rats were anaesthetized, the skin incised on the ventral abdominal midline and along the dorsal-lumbar aspect of the spine. The vagus nerve electrode array was tunneled subcutaneously from the dorsal-lumbar incision to exit through the ventral abdominal incision. The abdominal cavity was exposed and the liver retracted gently using sterile saline soaked gauze. Abdominal tissue was kept moist at all times using warm sterile saline. The sub-diaphragmatic anterior abdominal branch of the vagus nerve was dissected away from the esophagus and the array implanted rostral to the hepatic and celiac branches of the vagus (Figure 1A,C). The array was sutured (7-0 silk, Ethicon) to the esophagus to provide stabilization and the abdominal cavity and skin sutured closed. The rat was rotated to expose the dorsal aspect of the spine, the percutaneous connector was anchored to the connective tissue of the lumbar region of the spine, and the skin closed around it.

#### Electrode Impedance Testing and Electrophysiological Recordings

To test the functionality of electrodes, the impedance of electrodes was measured using biphasic current pulses passed between the electrode of interest and all other implanted electrodes (Fallon et al., 2009). The peak voltage at the end of the first phase (V_total_) of the current pulse was measured following delivery of a 25 μs per phase current pulse and current of 931 μA (Richardson et al., 2009). The V_total_ value was then used to calculate total impedance (*Z*_total_) using Ohm’s law (*Z* = voltage/current).

Electrically-evoked compound action potentials (ECAPs) were recorded in anaesthetized rats using bipolar vagus nerve electrodes. Either pair of electrodes (E1–E2 or E3–E4) could be used to stimulate or record neural responses. Two sets of evoked electrophysiological recordings (averaged from a total of 50 responses) were made at currents from 0 to 2 mA in 0.1 mA steps using a biphasic pulse (width = 200 μs, 50 μs interphase gap) presented at a rate of 10 pulses per second. Recordings were sampled at a rate of 100 kHz and filtered (high pass: 200 Hz; low pass: 2000 Hz; voltage gain 10^3^) (Payne et al., 2018a). The electrically-evoked neural response threshold was defined as the minimum stimulus intensity producing a response amplitude of at least 0.1 μV within a post-stimulus latency window of 4.0–7.0 ms (Payne et al., 2018a). All recorded neural responses had conduction velocities within the range of a C-fiber response (Castoro et al., 2011).

### VNS Off-Target Effects Experiment

Acute experiments (*n* = 5) were performed in normal, isoflurane anaesthetized, freely respiring rats to assess changes to heart rate, respiration rate and blood pressure during cervical and abdominal VNS. *Vagus nerve implantation surgery*: The left cervical vagus nerve was exposed and identified (Childs et al., 2015), and a vagus nerve electrode array (Figure 1B) implanted around the nerve. In the same rat, a second vagus nerve electrode array was implanted onto the anterior abdominal vagus nerve (see section above for details). *Femoral artery cannulation and measurements*: To measure arterial blood pressure changes, the femoral artery was exposed and cannulated. The cannula was connected to a calibrated blood pressure transducer (World Precision Instruments (WPI), Canada), the signal amplified and waveforms recorded (Cerebus System 128 Channel Neural Stimulator, Blackrock Microsystems, Massachusetts). *Heart and respiration rate measurements*: Heart rate was measured by recording electrocardiograms (ECG) by placing needles (26 Gauge) across the thorax and a return in the left leg. The ECG was amplified using a WPI Iso-80 bioamp (Gain: ×10^3^; high pass: 5 Hz; low pass 10 kHz) before being recorded via the Cerebus system. Respiration rate was measured by placing a PolyPower^®^stretch sensor (Danfoss PolyPower, Denmark) around the upper thorax. Care was taken to place the respiratory band sensor over the largest excursion point during respiration. *Stimulus-induced off-target testing*: Baseline recordings of heart rate, respiration rate or blood pressure were generated for 30 s during which no (cervical or abdominal) stimulation was applied. After baseline recordings were taken, the cervical or abdominal vagus nerve was stimulated (10 Hz, 50 repetitions) at 0 or 1.6 mA (200 μs pulse width) for 20 s. To confirm that 1.6 mA stimulation applied to the cervical or abdominal vagus nerve was suprathreshold, electrically-evoked neural responses were recorded (1.6 mA, 200 μs pulse width). Following this stimulation period, 30 s of recordings were taken to monitor the return of measurements to baseline. Heart rate, respiration rate and blood pressure changes from baseline were calculated from the waveforms using a detection algorithm in IGOR8 software. At the conclusion of the experiment, rats were euthanized. No tissue was taken for histology.

### VNS Efficacy Experiment

Chronic experiments (*n* = 15) assessed the efficacy of abdominal VNS in reducing inflammatory markers following TNBS-induced inflammation of the small intestine (experimental overview shown in Figure 1D).

#### Experimental Groups

The primary experimental groups consisted of rats that were implanted with an electrode array onto the abdominal vagus, allowed to recover for 7 days, habituated for 6 days and injected with TNBS to inflame the small intestine (see below). At 4 h following the TNBS injection, rats were randomly selected to receive abdominal VNS (TNBS+VNS: *n* = 7) or no stimulation (TNBS: *n* = 8). Blood and stool samples were collected, and ECAPs were recorded on days -14, 0, and 4 (Figure 1D). Rats were euthanized 4.5 days after TNBS injection (*T* = 4) and tissue taken for histological analysis. Similar to previous experiments (Pontell et al., 2009), control tissue (*n* = 9) was taken 5 cm oral to the ligation for the TNBS injection from animals in the TNBS (*n* = 5) and TNBS+VNS (*n* = 4) groups. An additional cohort of animals were euthanized 4 h after TNBS injection (4 H TNBS; *n* = 3) in order to evaluate the degree of intestinal inflammation at the onset of stimulation (Table 1).

**Table 1:** Experimental cohorts in the efficacy of VNS experiment.

Experimental group	TNBS	VNS	Sample size
TNBS only (4H TNBS)	Yes	N/A	3
Unstimulated (TNBS)	Yes	No	8
Stimulated (TNBS+VNS)	Yes	Yes	7

*Control tissue was taken from an oral segment of gut from implanted animals (control tissue from TNBS rats: n = 5 of 8; Control tissue from TNBS+VNS rats: n = 4 of 7).*

#### Inflammation of the Small Intestine

At 14 days following implantation of the electrode array onto the abdominal vagus nerve, rats were anaesthetized and under aseptic conditions the abdominal midline incised and an 8 cm segment of jejunum, clear of intra-luminal content, was selected and ligated between two sutures (2-0 silk, Ethicon; Nurgali et al., 2007). Inflammation was induced within this ligated area by slowly injecting 1 mL of TNBS (2.5% dilution in 50% ethanol, Sigma) at the oral end of the ligated small intestine over a course of 2 min. After 5 min the ligatures were removed, the intestine returned to the abdominal cavity and the skin and abdominal wall muscle sutured closed in two layers (Pontell et al., 2009). The small intestine was kept moist with sterile saline solution during the whole procedure.

#### Habituation and Vagus Nerve Stimulation

Rats were habituated for 6 consecutive days prior to the TNBS injection (*T* = -7 to -1) to ensure no additional stress to the animal during the testing period (*T* = 0–4). Animals were housed individually in Perspex boxes and percutaneous plugs connected to an external stimulator (Fallon et al., 2018), but no stimulation was delivered. Given that routine laboratory procedures can cause stress to the animal, every attempt to handle animals equally and as little as possible was made (Balcombe et al., 2004). Immediately prior to the TNBS injection (*T* = 0), ECAPs were generated in all implanted animals. At 4 h following TNBS injection, awake animals were randomly selected to receive VNS delivered at 1.6 mA and 200 μs/phase (i.e., 320 nC per phase) using a stimulus rate of 10 pulses/s with a 30 s ON 5 min OFF duty cycle for 3 h/day (1:30–4:30 p.m.) for 5 consecutive days (*T* = 0 to *T* = 4; 5 stimulation sessions in total). Unstimulated rats (TNBS group) were subjected to the same procedures as stimulated rats, but did not receive VNS. ECAPs were recorded on days -14, 0, and 4 (Figure 1D). Electrical stimulation was delivered using an external battery operated stimulator (Fallon et al., 2018) connected to the percutaneous connector. The stimulator delivered charge-balanced biphasic current pulses to the selected bipolar electrodes located on the nerve. Charge recovery was achieved via electrode shorting on completion of each current pulse.

#### Quantification of Disease Activity Index

Stool produced from implanted rats while being weighed (between 9 and 10 a.m. each day (*T* = 0 to *T* = 4) was assessed for consistency and signs of bleeding (Table 2 and Figure 1D) (Sun et al., 2013).

**Table 2:** Scoring system of stool quality following TNBS injection.

Variable	0	1	2
Stool consistency	Normal stool: hard pellet shaped form	Loose stools: Pellet is sticky and deforms under pressure	Diarrhea: No form; fecal matter adherent to fur
Signs of blood	No blood: Stool is a medium brown color	Mild bleeding: Stool is dark brown or black in appearance	Gross bleeding: Blood is visible on fur and bedding

#### Quantification of C-Reactive Protein

Blood was taken from the tail vein of implanted rats (Figure 1D). Blood taken prior to TNBS injection on day 0 served as a control. The final bleed was taken after the final round of stimulation. Whole blood (300 μL) was collected in K2-EDTA tubes (Starstedt) centrifuged (2000 g for 10 min) and plasma aliquoted and stored at -80°C. On the day of the assay, aliquots were thawed on ice and the C-reactive protein (CRP) ELISA conducted according to manufacturer instructions (Cusabio CSB-E07922r) and CRP levels determined via absorbance measurements using a Biorad BenchMark Plus microplate spectrophotometer.

#### Dissection, Histology, and Immunohistochemistry of Small Intestine Tissue

At the conclusion of the experiment, implanted rats were euthanized and the TNBS-inflamed segment of small intestine tissue dissected and processed as previously described (Payne et al., 2018c). In brief, a 2 cm segment of control tissue was removed spanning 5–7 cm oral to where the ligation limiting the inflamed site had been. As TNBS-induced inflammation is patchy, the 8 cm length of inflamed intestine was divided equally into four 2 cm long segments, and cut longitudinally along the mesenteric border and pinned out onto balsa boards (mucosa side up). One half was placed in fixative (2% formaldehyde plus 0.2% picric acid in 0.1 M sodium phosphate buffer, pH 7.4) overnight, embedded in paraffin, sectioned (5 μm) and stained with hematoxylin and eosin (H&E) (Pontell et al., 2009) or immunohistochemically stained with anti-CD3, a cytotoxic T cell marker (1:200; Cytomation, Dako E0432) (Payne et al., 2018c). The other half of the tissue was processed for frozen sections (14 μm) and myeloperoxidase (MPO) staining (Payne et al., 2018c). All sections were mounted with DPX.

#### Histopathology Scoring of Inflamed Tissue

Histopathologist (J.B.Furness), blinded to procedures, used H&E stained sections to evaluate the degree of inflammation in each segment of intestine. Histological changes were on a scale of 0–3 for the assessment of damage to the mucosa (villi architecture changes, including loss of height, pinching, clubbing, venous engorgement), and assessment of inflammatory changes (leukocyte infiltration) on a scale of 0–2 for assessment of the numbers of leukocytes within venules (adapted from Payne et al., 2018c; Table 3). Scores were out of a total of 9.

**Table 3:** Histological parameters used to score tissue taken from the small intestine following TNBS injection.

	Variable	0	1	2	3
Mucosal damage	Extent of mucosal damage (including loss of height, pinching, clubbing, venous engorgement)	No damage	Damage affects less than 1/3 of villi	Damage affects between 1/3 and 2/3 of villi	Damage affects more than 2/3 of villi
	Shortening of villi	0–20% shortening	20–60% shortening	60–100% shortening	N/A
	Pinching of villi	Absent	Affecting < 50% of villi	Affecting > 50% of villi	N/A
Inflammatory changes	Leukocyte presence in large venules (avoiding capillaries and small venules or lymphatics)	<4 adherent leukocytes in venules	4–10 adherent leukocytes in venules	>10 adherent leukocytes in venules	N/A

*Adapted from Payne et al. (2018c).*

#### Inflammatory Cell Quantification in Transmural Small Intestine Tissue

Eosinophils, T cells (CD3+ cells) and neutrophils (MPO+ cells) were quantified using a Zeiss Axioplan II microscope, positive cells were counted (×40 objective) across three consecutive fields of view across the external smooth muscle layers, submucosal and mucosal layers. Cells were counted within the most inflamed area of the tissue. Images of the total field of view were generated (Axiovision, Zeiss, Germany). For MPO, eosinophil and T cell counts, cells per mm length of intestine were quantified.

#### Vagal Nerve Tissue Processing and Analysis

Immediately following dissection of small intestine tissue, implanted animals were perfused intracardially with 0.9% saline followed by fixative (4% paraformaldehyde in 0.1 M phosphate buffer, pH 7.4, room temperature). The esophagus and implanted vagus nerve array were dissected from the carcass. At the implanted region, the vagus nerve was dissected from the array and the region of the nerve adjacent to the electrodes (E1–E4) labeled using tissue dye (Davidson’s Marking system, Bradley Products, MN, United States) (Villalobos et al., 2013). Tissue proximal to the implanted site was also taken and processed as an intra-animal, non-implanted control. The esophagus and attached vagus nerve were embedded in paraffin and serial sections (5 μm) taken. The tissue dye marked the area of vagus nerve that was adjacent to electrodes. Sections were stained for H&E and mounted with DPX. Sections were examined by an observer (S.C Payne), blinded to procedures, for signs of histopathological damage. At each location light microscope images were taken using a Zeiss Axioplan II microscope and Axiovision software (Zeiss, Germany). Using ImageJ, total fascicle area was quantified across one section per electrode position, per animal. The cross-sectional area of the vagus nerve was not measured as the boundary of the epineurium was difficult to define.

#### Statistics

Differences between normally distributed data were tested using a one- or two-way repeated measures (RM) ANOVA with Sidak or Tukey *post hoc* tests as appropriate. Differences between data that was not normally distributed was analyzed using a non-parametric Kruskal Wallis one-way ANOVA and Dunn’s *post hoc* test. Details of each statistical test are stated in the relevant results section. Statistically significant differences were accepted as *P*-values of < 0.05 and GraphPad Prism 4 (GraphPad Software, United States) was used for all analysis.

## Results

### VNS Off-Target Effects Experiment

#### No Measurable Off Target Affects During Abdominal Vagus Nerve Stimulation

**FIGURE 2:**
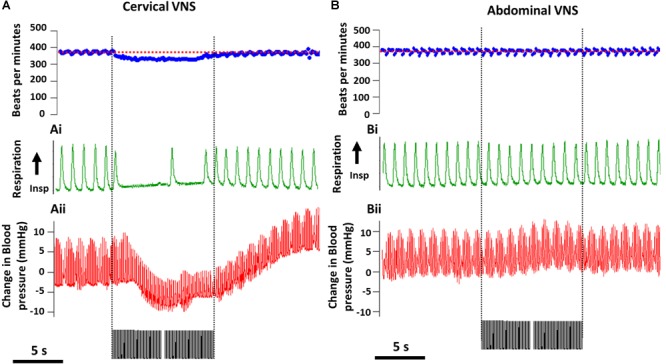
Off-target effects during cervical and abdominal VNS. Representative recordings from from one animal show a decrease in heart rate (baseline heart rate indicated by red dotted line) **(A)**, respiratory rate **(Ai)** and blood pressure **(Aii)** during cervical VNS (1.6 mA, 200 μs/phase). **(B–Bii)** No changes in heart rate (baseline heart rate indicated by red dotted line) **(B)**, respiratory rate **(Bi)** and blood pressure **(Bii)** were seen during abdominal VNS (1.6 mA, 200 μs/phase). Scale bar in **(A,B)** represents 5 s.

The average (standard error of mean, SEM) threshold for activation of C-fibers by cervical VNS was 0.25 ± 0.07 mA and abdominal VNS was 0.43 ± 0.11 mA, indicating the test stimulation of 1.6 mA was substantially suprathreshold for C-fibers at both stimulation sites in all animals. In the example shown in Figure 2A, cervical VNS resulted in an average heart rate drop of 43 beats per minute (bpm) from baseline (370 ± 15.4 bpm), a maximum decrease in blood pressure of 8.2 mmHg and an almost complete cessation of breathing (baseline respiration rate: 52 ± 7.0 cycles per minute, cpm). In contrast, the same level of abdominal VNS produced no change in heart rate (400 ± 31.4 bpm), repiration rate (54 ± 5.0 cpm) or blood pressure (Figure 2B).

Statistical analysis [two-way (Current × Location) RM ANOVA; *n* = 5] of the effects of VNS on heart rate revealed a significant effect of Current (*P* = 0.06), Location (*P* = 0.04) and a significant Interaction (*P* = 0.02). Tukey’s *post hoc* analysis showed the average 31 ± 9 bpm (mean ± SEM) drop in heart rate during suprathreshold cervical VNS was significantly (*P* = 0.02) greater than suprathreshold abdominal VNS, which was not different to no stimulation (*P* = 0.9). Respiration recordings were noisy and difficult to quantify, however, severe disruptions to the regular respiration pattern were observed in 4 of 5 rats during the suprathreshold cervical VNS, while no changes in respiration were observed with abdominal VNS. Blood pressure changes were only assessed in *n* = 2 animals and therefore no statistical comparisons of the data were performed.

### VNS Efficacy Experiment

#### Thresholds for Electrically-Evoked Neural Responses Remained Below Stimulation Levels

**FIGURE 3:**
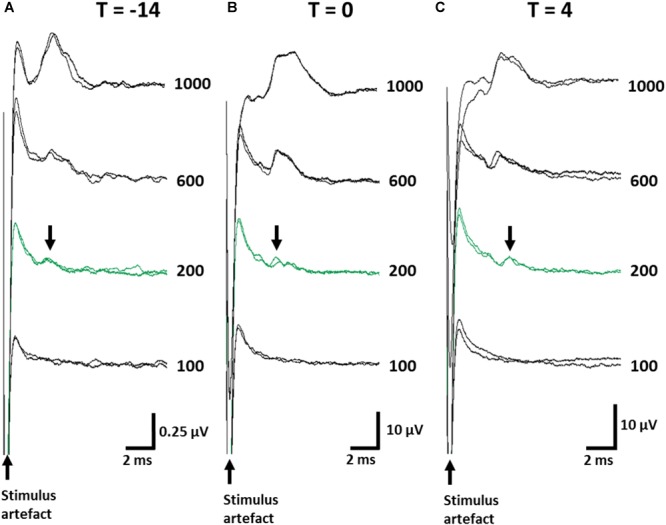
Electrically-evoked C-fiber responses from the abdominal vagus nerve were recorded over the implantation period. Representative ECAPs from one animal are shown when taken immediately following implantation surgery (**A**, *T* = -14), prior to the injection of TNBS (**B**, *T* = 0) and at the conclusion of the experiment (**C**, *T* = 4). Note, the amplitude of the neural response recorded immediately post-implantation (*T* = -14) were consistently smaller than those at later time points green traces indicate the neural threshold (200 μA) and arrows indicate the elecrically evoked C-fiber response (Latency range: 5.3–5.6 ms). Current amplitude (in μA) is indicated to the right of each set of ECAPs.

ECAPs were recorded to ensure stimulation levels were above neural threshold. The mean threshold of recorded ECAPs remained unchanged (one-way RM ANOVA (Time): *P* = 0.8) between day -14 (393 ± 74 μA; Figure 3A), day 0 (357 ± 65 μA; Figure 3B) and day 4 (325 ± 75 μA; Figure 3C) and substantially below the levels used to delivery therapeutic stimulation (1.6 mA, 200 μs/phase). The latency of ECAPs also remained unchanged (one-way RM ANOVA; *P* = 0.16) during the implantation period (*T* = -14: 5.43 ± 0.35 ms; *T* = 0: 6.69 ± 0.58 ms; *T* = 4: 5.52 ± 0.35 ms).

#### Impedance of Implanted Abdominal Vagus Nerve Electrodes

The mean electrode impedance (±SEM) in saline (prior to implantation) was 2308 ± 96 Ω (range: 1880–3340 Ω). Immediately following implantation, common ground impedance of electrodes increased to 5070 ± 246 Ω (range between 3485 and 7194 Ω). At the conclusion of the experiment the *in vivo* impedances had increased to 8379 ± 269 Ω (5807–9650 Ω). During the implantation period, there were no short circuits, and only 4 out of 60 electrodes (*n* = 4 electrodes per rat; *n* = 15 implanted rats in total) became open circuit. These short circuits did not compromise the delivery of VNS.

#### Vagus Nerve Stimulation Improved Clinical Measurements of Inflammation

**FIGURE 4:**
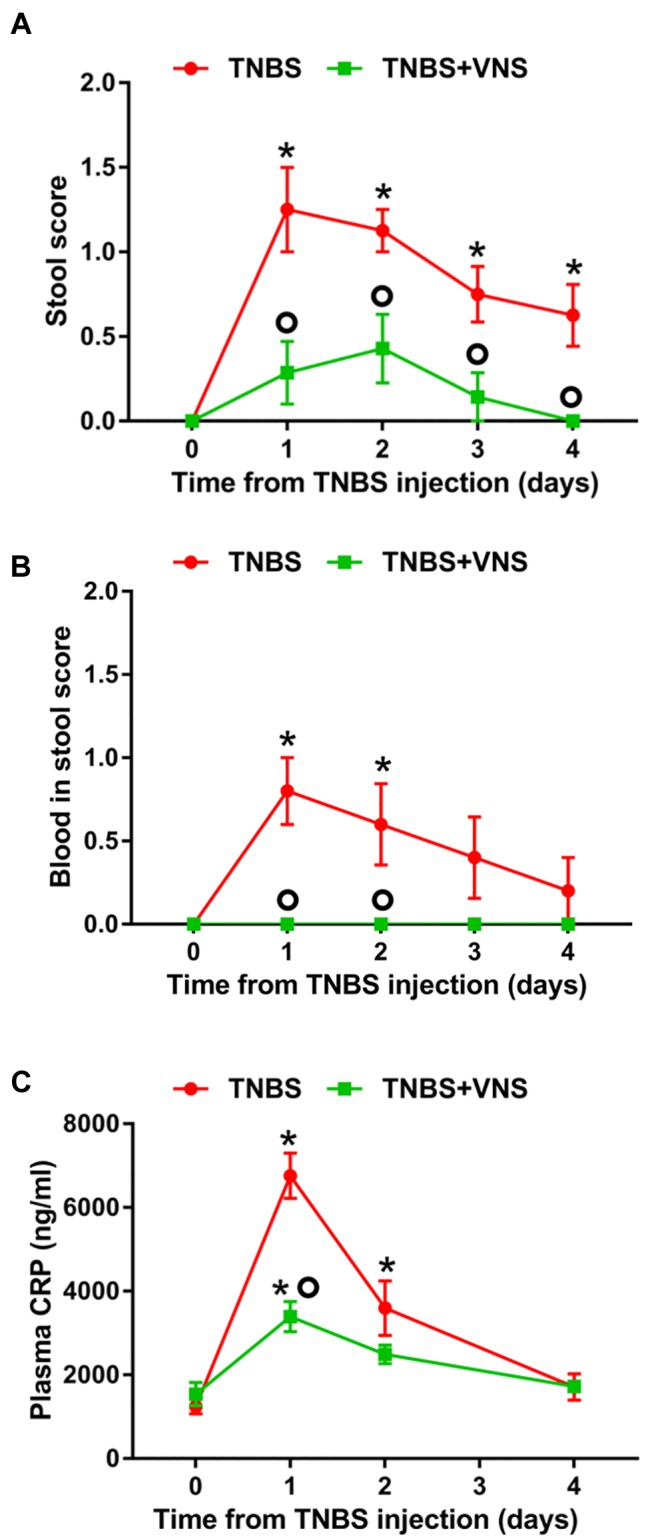
**(A)** Vagus nerve stimulation improves clinical measurements of inflammation following TNBS injection. **(A)** The quality of stool produced from unstimulated (TNBS; *n* = 8) and stimulated (TNBS+VNS; *n* = 7) rats were scored following the TNBS injection. **(B)** Blood in stool produced from unstimulated (TNBS; *n* = 5) and stimulated (TNBS + VNS; *n* = 4) rats was scored following TNBS injection. **(C)** CRP content in blood plasma was measured in unstimulated (TNBS; *n* = 6) and stimulated (TNBS+VNS; *n* = 6) on day 0 (pre-TNBS control) and days 1, 2, and 4 following TNBS injection. All data show mean ± SEM. Significant differences from control (day 0, pre-TNBS) are indicated by “^∗^”, while significant differences between unstimulated (TNBS) and stimulated (TNBS+VNS) groups are indicated by “O”.

On day 0 (referred to as the “control”), prior to the TNBS injection, all implanted rats (*n* = 15) produced stools that were solid, dry pellets, with no signs of blood. Following TNBS injection, unstimulated rats (TNBS, *n* = 8) had significantly worse stool quality [two-way RM ANOVA (Time × Treatment): Time: *P* < 0.0001; Treatment *P* = 0.0009; Interaction: *P* = 0.02] than control (day 0) on day 1 (*Sidak post hoc* test: *P* < 0.0001), day 2 (*P* < 0.0001), day 3 (*P* = 0.002) and day 4 (*P* = 0.015; differences indicated by “^∗^” in Figure 4A). The stool quality of stimulated rats (TNBS+VNS, *n* = 7) remained similar to control following TNBS injection (*P* ≥ 0.05). Furthermore, stimulated rats (TNBS+VNS) had significantly better stool quality than unstimulated rats (TNBS) on day 1 (*P* = 0.0002), day 2 (*P* = 0.012), day 3 (*P* = 0.04), and day 4 (*P* = 0.03; differences indicated by a ‘circle’ in Figure 4A).

Following TNBS injection, the presence of blood in feces was observed (two-way RM ANOVA: Time: *P* = 0.058; Treatment *P* < 0.0001; Interaction: *P* = 0.058) in unstimulated rats (TNBS; *n* = 5) significantly more often, compared to control (day 0), on days 1 (*P* = 0.0002) and day 2 (*P* = 0.006; differences indicated by “^∗^” in Figure 4B).

Unstimulated rats (TNBS; *n* = 5) were observed to have higher presence of blood in feces than stimulated rats (TNBS+VNS) on day 1 (*P* = 0.001) and day 2 (*P* = 0.02; differences indicated by a “circle” in Figure 4B). Stimulated rats (TNBS+VNS; *n* = 4) were not observed to have blood in feces at any time point following TNBS injection (Figure 4B).

Following TNBS treatment, plasma CRP levels in unstimulated rats (TNBS) were significantly increased [two-way RM ANOVA (Stimulation × Time); Treatment: *P* = 0.005; Time, *P* < 0.0001, Interaction, *P* < 0.0001] at day 1 (Sidak *post hoc*: *P* < 0.0001) and day 2 (*P* = 0.0004), compared to day 0 (indicated by “^∗^” in Figure 4C). By day 4, CRP levels returned to baseline levels. CRP levels of stimulated rats (TNBS+VNS) were significantly higher than baseline only at day 1 (*P* = 0.006), and no different from control (day 0) on days 2 and 4 (*P* ≥ 0.05). However, on day 1 CRP levels were still significantly lower (*P* < 0.0001) than unstimulated rats (TNBS, indicated by a “circle,” Figure 4C).

#### TNBS-Induced Inflammatory Damage Improved Following VNS

**FIGURE 5:**
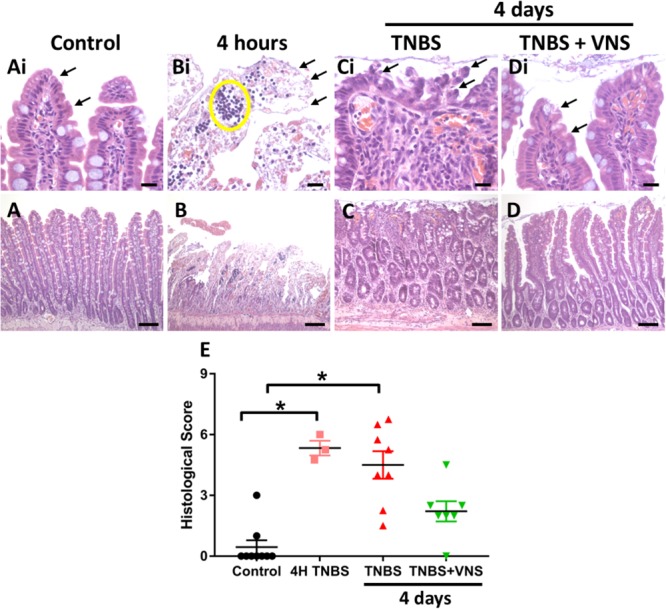
Vagal nerve stimulation reduced histological damage of small intestine tissue following TNBS injection. **(A)** Control small intestine **(A,Ai)** had undamaged, intact surface epithelium (arrows, **Ai**). **(B,Bi)** At 4 h following TNBS injection (the time that VNS was applied), extensive epithelial cell loss (arrow, **Bi**) and leukocyte infiltration (yellow circle) was observed. **(C,Ci)** At 4 days following TNBS injection, there was extensive damage to villus architecture (**Ci**, arrows) and leukocyte infiltration in venules (circle) in tissue from unstimulated rats (TNBS). **(D,Di)** In tissue from stimulated rats (TNBS+VNS), histological damage was less severe than tissue taken from unstimulated (TNBS) rats. Surface epithelial cells were intact (arrows), and villi were mostly undamaged, although some inflammation-induced irregularities remained. **(E)** The histological score was quantified in tissue taken from control animals, at 4 h following TNBS injection, and from unstimulated (TNBS) and stimulated animals (TNBS+VNS). Data show raw histological score (0–9) of each animal, median and interquartile range. Significant differences of *P* < 0.05 are indicated by “^∗^.” Scale bars in **(A–D)**: 100 μm; in **(Ai–Di)**: 20 μm.

In small intestine tissue 5–7 cm oral to the site of TNBS induced inflammation (i.e., control tissue), there was no damage to the epithelial cells of the villi along the length of the analyzed tissue, with villi exhibiting the normal long finger-like projections (Figure 5A,Ai). Few leukocytes (less than 4) were observed within venules of the lamina propria and within the circular and longitudinal muscle layers, while there was mild infiltration of leukocyte populations within the mucosa and submucosa layers. In acute TNBS tissue (4 h post-injection, at the time that active VNS was applied) extensive epithelial cell loss (Figure 5B,Bi indicated by arrows) and leukocyte infiltration (Figure 5B,Bi, indicated by circle) was observed. Leukocyte infiltration into the mucosa/submucosal layers was moderate. At 4 days following TNBS injection, tissue from unstimulated (TNBS) rats had extensive irregularities to villus epithelial cells (Figure 5C,Ci, indicated by arrows). Villi were shorter and had a blunted appearance, and leukocyte infiltration was severe within the mucosa, submucosa and venules (Figure 5C,Ci). Tissue from stimulated (TNBS+VNS) animals exhibited reduced inflammation-induced damage. Less damage or abnormalities to villi surface epithelium was observed (Figure 5D,Di), while villi architecture was sometimes indistinguishable from control small intestine tissue. However, some inflammation-induced irregularities, such as shortening of villi remained within tissue from stimulated animals (TNBS+VNS; Figure 5D).

The histological score measured from control tissue of unstimulated (TNBS; *n* = 5) and stimulated (TNBS+VNS; *n* = 4) rats were no different from each other (*P* ≥ 0.05; unpaired *T*-test). Therefore the control groups were combined (*n* = 9). Statistical analysis of the histological score showed there was significant (Kruskal–Wallis one-way ANOVA: *P* = 0.001) histological damage in tissue from 4 h post-TNBS injection (Dunn’s *post hoc*: *P* = 0.002) and in unstimulated rats (TNBS; *P* = 0.0006; Figure 5E). However, the histological score of tissue taken from stimulated animals (TNBS+VNS) was no different from control (*P* = 0.17), suggesting that VNS improved recovery from inflammation-induced damage (Figure 5E).

#### Numbers of Leukocytes in Mucosal and Submucosal Layers Was Reduced Following VNS

**FIGURE 6:**
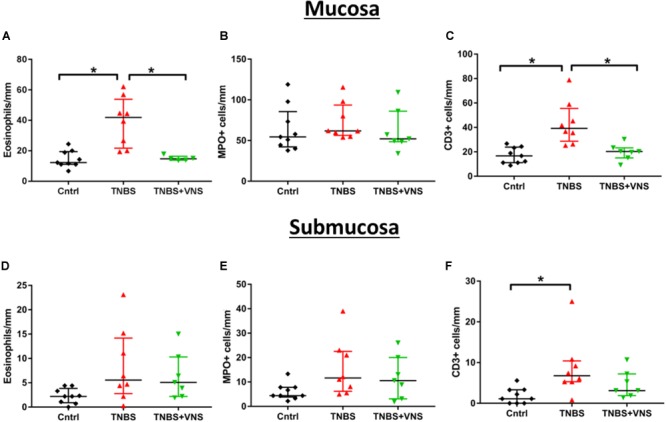
VNS reduces resident leukocyte populations in mucosal and submucosal layers following TNBS injection. **(A–C)** Quantification of eosinophils **(A)**, MPO+ cells (neutrophils, **B**) and CD3+ cells (T cells; **C**) within the mucosal layer. **(D–F)** Quantification of eosinophils **(D)**, MPO+ cells **(E)** and CD3+ cells **(F)** within the submucosal layer. Data show raw data from each animal, median and interquartile range. Significant differences of *P* < 0.05 are indicated by “^∗^.”

The numbers of leukocytes measured from the control mucosal, submucosal and muscle layers were no different from each other (*P* ≥ 0.05; unpaired *T*-test). Thus the control groups were combined (*n* = 9). Tissue taken 4 h (4 H TNBS; *n* = 3) following TNBS is shown in Figure 5B,Bi, but was not included in the statistical cell count analysis, nor included in subsequent graphs (Figure 6), due to the low number of animals in this group. Four days following the TNBS injection, there was a significant increase in eosinophils within the mucosal layer (Kruskall–Wallis: one-way ANOVA: Treatment *P* = 0.0002), and a significant increase in the number of CD3+ cells (T cells) within both the mucosa (*P* = 0.001) and submucosa (*P* = 0.01) in unstimulated animals (TNBS, Figure 6A–C). In contrast, there was no significant elevation in the number of eosinophils, MPO+ cells or CD3+ cells in stimulated animals (TNBS+VNS; Figure 6A–C). Furthermore, eosinophil (Dunn’s *post hoc*: *P* = 0.03) and T cells (*P* = 0.02) populations were significantly reduced in the mucosal layer of stimulated animals (TNBS+VNS), compared to unstimulated animals (TNBS; Figure 6A,C).

#### Leukocyte Infiltration Into External Muscle Layers Was Reduced Following VNS

**FIGURE 7:**
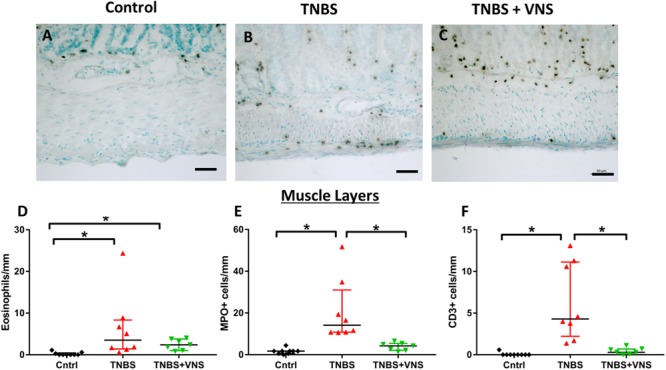
VNS reduces resident leukocyte populations in muscle layers following TNBS injection. **(A–C)** Images show myeloperoxidase (MPO) positive cells (neutrophils) within circular and longitudinal muscle layers of tissue taken from control **(A)**, TNBS rats **(B)** and TNBS+VNS rats **(C). (D–F)** Quantification of eosinophils **(D)**, MPO+ cells **(E)**, and CD3+ cells **(F)** within the circular and longitudinal muscle layers. Data show mean count/animal, median and interquartile range. Significant differences of *P* < 0.05 are indicated by “^∗^.”

Tissue taken 4 h (4 H TNBS; *n* = 3) following TNBS was not included in the statistical cell count analysis, nor included in subsequent graphs (Figure 7), due to the low number of animals in this group. In the circular and longitudinal muscle layers, there was an increase in eosinophils within tissue taken from unstimulated (TNBS; Kruskal–Wallis: *P* = 0.001) and stimulated rats (TNBS+VNS; *P* = 0.012), compared to control (Figure 7D). Numbers of MPO+ cells (representative images for control, TNBS and TNBS+VNS tissue shown in Figure 7A–C) (neutrophils) increased in tissue taken from unstimulated rats (TNBS; *P* < 0.0001), but was significantly less in tissue taken from stimulated rats (TNBS+VNS; *P* = 0.048; Figure 7E). Numbers of CD3+ cells (T cells) within the muscle layers of tissue taken from TNBS treated rats were greater than control (*P* < 0.0001). However, there were significantly fewer T cells in the muscle layer of tissue taken from stimulated rats (*P* = 0.04), compared to unstimulated rats treated with TNBS (Figure 7F).

#### Assessment of the Implanted Vagus Nerve

**FIGURE 8:**
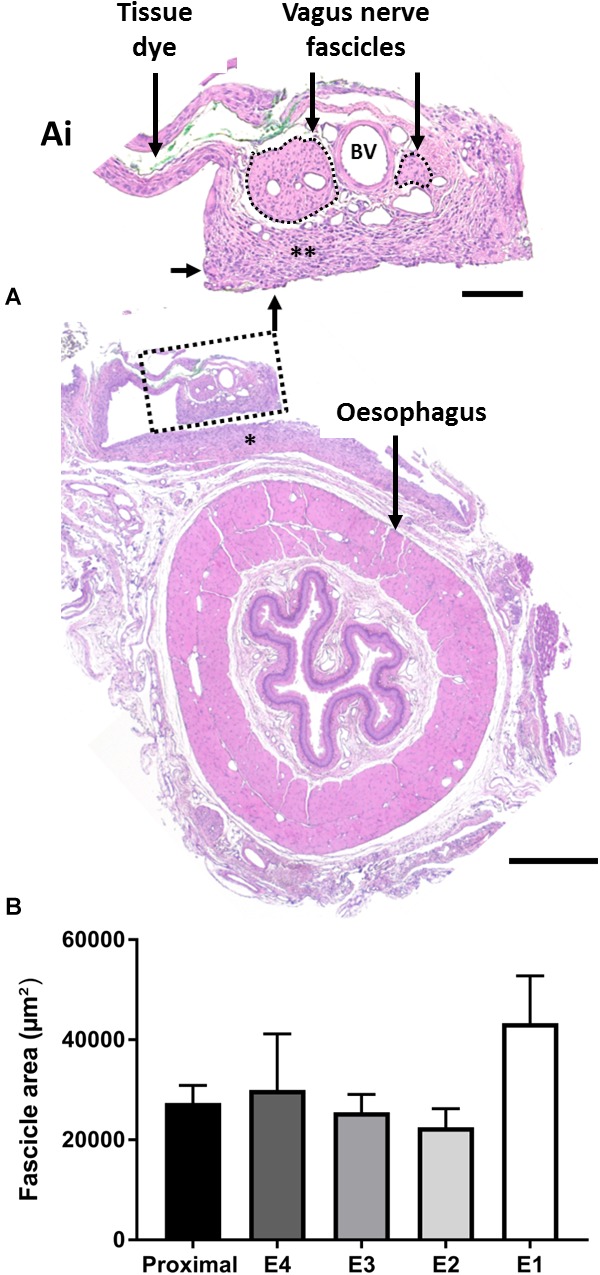
Histopathology of the anterior abdominal vagus nerve following implantation. **(A,Ai)** H&E stained vagus nerve that was stimulated for 4 days shows a benign foreign body tissue response (**A**, indicated by ^∗^), which extended into the electrode channel (**Ai**, indicated by ^∗∗^). There were no acute inflammatory cells within the epineurium, the two fascicles of the vagus nerve (outlined by a dotted line) appeared undamaged, and blood vessels (example indicated by “BV”) appeared patent. Tissue dye was used to identify the implanted nerve upon dissection. **(B)** Quantification of total fascicle area (example indicated by dotted lines in **Ai**) between non-implanted neural tissue, taken proximal to implantation site, and implanted tissue adjacent to electrodes E1–E4. Data show mean fascicle area (μm^2^) + SEM, and there were no significant differences between locations. Scale bar in **(A)** 500 μm Scale bar in **(Ai)** 100 μm.

Chronically implanted abdominal vagus nerves (*n* = 9) were assessed for histopathological changes (Figure 8A,Ai) and changes in fascicle area (Figure 8B). We observed no tissue granulation or infiltration of acute inflammatory cells, however, a foreign body tissue response (Figure 8Ai, indicated by ^∗∗^) was seen within the electrode channel, surrounding the nerve (Figure 8Ai). No inflammatory cells were observed suggesting the foreign body response was likely benign. Furthermore, blood vessels (Figure 8Ai, indicated as “BV”) were observed within the nerve. There were no observed differences between stimulated and unstimulated nerve histology.

The fascicle area (indicated by dotted lines, Figure 8Ai) of the nerve adjacent to the electrodes E1-E4 was analyzed and compared to non-implanted vagus nerve tissue (taken proximal to the implantation site). There were no significant changes in fascicle area between implanted (E1–E4) and non-implanted (proximal) tissue (one-way RM ANOVA: *P* = 0.9; *n* = 9) (Figure 8B).

## Discussion

VNS was effective in improving a number of markers of inflammation, including stool quality, systemic inflammation and leukocyte infiltration within the small intestine. Furthermore, significant off-target cardiac and respiratory events occurred during suprathreshold cervical VNS, while in contrast, no measured off-target changes were seen during abdominal VNS. The absence of off-target effects and efficacy in reducing inflammation suggests that abdominal VNS is a suitable alternative to cervical VNS. Taken together, these findings support the use of this novel peripheral nerve array for abdominal VNS as a potential treatment for IBDs, such as Crohn’s disease.

Stimulation of the cervical vagus nerve at higher frequencies (30 Hz) or lower frequencies (10 Hz) has the potential to activate fibers to the larynx, heart and lungs, in addition to the gastrointestinal tract and liver (Bonaz et al., 2013). In this study, cervical VNS (10 Hz, 1.6 mA, 200 μs) caused a decrease in heart rate and respiration. These changes can be attributed to the activation of large pulmonary stretch A-fibers and cardio-inhibitory vagal B fibers (McAllen et al., 2018). In a retrospective clinical study, a significant proportion of epileptic patients (*n* = 95) receiving cervical VNS (30 Hz, 0.25–3.5 mA, 500 μs) treatment reported stimulation-induced hoarseness of voice (63%), coughing (44%), and pain (37%) (Handforth et al., 1998; DeGiorgio et al., 2000). Similarly, ileocecal Crohn’s disease patients treated with cervical VNS (10 Hz, 500 μs, 1.25 mA) also reported dysphonia during stimulation, although symptoms resolved over time (Bonaz et al., 2016). In humans, the effects on heart rate are less pronounced than that reported in rats, nevertheless, some clinical studies show disruptive effects to heart contractility and heart rate during stimulation of the left cervical and thoracic vagus nerve, i.e., above the vagal cardiac branches (Frei and Osorio, 2001; Lewis et al., 2001). Taken together, cervical VNS has a number of unwanted, albeit often mild, off-target effects. Such off-target effects potentially limit the intensity and duration of therapeutic stimulation delivered, thereby potentially compromising or limiting the effectiveness of the bioelectric neuromodulation treatment. In contrast abdominal VNS did not evoke changes in heart rate, respiration rate or blood pressure. These findings are in agreement with previous reports of abdominal VNS in anaesthetized pigs and humans for the treatment of post-operative ileus (Stakenborg et al., 2017), and is consistent with the known functional anatomy of the vagus.

Engaging the optimal nerve fiber population is essential for an effective bioelectric neuromodulation therapy (Payne et al., 2018b). The cervical vagus nerve consists of a mixed population of large, myelinated A-fibers, smaller myelinated B-fibers and small, unmyelinated C-fibers, which have different electrical activation thresholds (Castoro et al., 2011). This is problematic for cervical VNS for the treatment of intestinal inflammation as activation of vagal C-fibers, thought to be involved in driving anti-inflammatory effects in the intestine (Martelli et al., 2014; Payne et al., 2018b), typically also activates A-fibers (Castoro et al., 2011; McAllen et al., 2018). Selective activation of C-fibers, while minimizing the activation of A- and B-fiber populations is very difficult to achieve long-term *in vivo* (reviewed in Guiraud et al., 2016). Some studies have demonstrated a partial preferential activation of C-fibers while suppressing A-fibers activation in the vagus nerve of pigs by delivering a non-rectangular pulse waveform (Tosato et al., 2007). However, this stimulation strategy requires high, often unsafe levels of charge to be delivered in order to be effective (Vuckovic et al., 2008; Qing et al., 2015). Targeting the abdominal vagus nerve overcomes this issue as the nerve consists primarily of C-fibers (rats: 99%; humans: 97%) (Hoffman and Schnitzlein, 1961; Prechtl and Powley, 1990). Furthermore, targeting this C-fiber dense segment of the vagus nerve might increase the clinical efficacy of stimulation in electrically-activating C-fibers. We measured the conduction velocity of the electrically-evoked neural responses elicited by abdominal VNS and they ranged between 0.55 and 0.84 m/s, which fits within the range of C-fiber conduction velocities (Castoro et al., 2011).

Abdominal VNS was effective in reducing TNBS-induced inflammation, as indicated by changes in several markers, including the stool quality, systemic CRP, histology and leukocyte infiltration into transmural layers. In contrast, previous studies on experimental colitis showed cervical VNS had no protective effects within the inflammatory lesion site (Meregnani et al., 2011), and all measured inflammatory markers of colitis (mucosal index damage index, disease activity index, histological score and colonic cytokine content) remained significantly higher in tissue taken from cervical VNS rats, compared to control (*P* ≥0.05) (Sun et al., 2013). One explanation for this disparity is the differences in TNBS injections between studies. The concentration of TNBS administered here was 2.5% w/v, while others used higher concentrations of 40% (Sun et al., 2013) and ∼180% (Meregnani et al., 2011). Additionally, we injected TNBS into the small intestine, rather than the colon. Vagal innervation of the small intestine, specifically the jejunum, is denser than in the distal colon (Berthoud et al., 1990); therefore the therapeutic action of VNS may have had an increased potency.

The stimulation regime used in this study involved a charge density of ∼1 μC/cm^2^/phase, delivered for 3 h at the same time each day (1:30–4:30 p.m.), with a duty cycle of 30 s ON, followed by 5 min OFF. This stimulation is well below the safety limit for platinum electrodes (Cogan et al., 2016). The stimulation timing and duty cycle were chosen to be similar to those used in the treatment of colitis (Sun et al., 2013) and ileocecal Crohn’s disease (Bonaz et al., 2016), which in turn were based on VNS to treat patients with drug resistant epilepsy and depression (Sackeim et al., 2001; Cukiert et al., 2013). Therefore, it is quite likely that the timing and duty cycle used may not be optimal for the treatment of IBD; the optimization of stimulation parameters to those most appropriate for therapy has been poorly explored and remains a significant challenge for bioelectric neuromodulation therapies (Payne et al., 2018b). Testing of parameters, such as pulse durations, stimulation rate, duty cycles, and the duration and time of day that stimulation is applied, are essential to explore in appropriate animal models of the disease if bioelectric neuromodulation is to be successful long-term clinical treatment.

## Conclusion

We have developed a stimulating and recording electrode array that effectively activates C-fibers in the abdominal vagus nerve, allowing for VNS to treat inflammation following TNBS injection to be applied closer to the end organ with no measurable off-target effects. Our work supports abdominal VNS as an effective approach to the treatment of IBD in humans.

## Ethics Statement

All animal procedures were approved by the Animal Research and Ethics Committee of the Bionics Institute and complied with the Australian Code for the Care and Use of Animals for Scientific Purposes (National Health and Medical Research Council of Australia). Approval was also obtained from the United States Army Medical Research and Material Command Animal Care and Use Review Office, protocol SSC-7486.02.

## Author Contributions

All authors listed have made a substantial, direct and intellectual contribution to the work, and approved it for publication.

## Conflict of Interest Statement

The authors declare that the research was conducted in the absence of any commercial or financial relationships that could be construed as a potential conflict of interest.
